# Amelioration of Hyperglycaemia, Oxidative Stress and Dyslipidaemia in Alloxan-Induced Diabetic Wistar Rats Treated with Probiotic and Vitamin C

**DOI:** 10.3390/nu8050151

**Published:** 2016-05-05

**Authors:** Tagang Aluwong, Joseph O. Ayo, Alkali Kpukple, Olusola Olalekan Oladipo

**Affiliations:** 1 Department of Veterinary Physiology, Ahmadu Bello University, c/o P.O. Box 216 Samaru, 810006 Zaria, Nigeria; ayojo94@yahoo.com (J.O.A.); alkalivet@gmail.com (A.K.); 2 Biochemistry Division, National Veterinary Research Institute, 930103 Vom, Nigeria; oladiposola@ymail.com

**Keywords:** dyslipidaemia, hyperglycaemia, oxidative stress, type 1 diabetes, wistar rats

## Abstract

Clinical and experimental evidence suggests that hyperglycaemia is responsible for the oxidative stress in diabetes mellitus. The study was designed to investigate the comparative effects of probiotic and vitamin C (Vit-C) treatments on hyperglycaemia, oxidative stress and dyslipidaemia in alloxan-induced diabetic rats. Type 1 diabetes (T1DM) was induced in male Wistar rats by a single intraperitoneal (i.p.) injection of alloxan (150 mg/kg). Six groups of the animals received the following treatment regimens for four weeks: (1) Normal saline, *per os*; (2) alloxan (150 mg/kg, i.p.); (3) alloxan (150 mg/kg) + insulin (4 U/kg, subcutaneously); (4) alloxan (150 mg/kg) + probiotic (4.125 × 10^6^ CFU/100 mL *per os*); (5) alloxan (150 mg/kg) + Vit-C (100 mg/kg, i.m.); (6) alloxan (150 mg/kg) + probiotic (4.125 × 10^6^ CFU/100 mL *per os*) + Vit-C (100 mg/kg, intramuscularly). Probiotic + Vit-C decreased (*p* < 0.05) blood glucose concentration in diabetic treated group, when compared with the untreated diabetic group. Probiotic + Vit-C reduced malondialdehyde concentration, in the serum, brain and kidneys, respectively, but increased the activity of antioxidant enzymes. Probiotic and Vit-C may be more effective than Vit-C alone, in ameliorating hyperglycaemia, oxidative stress and dyslipidaemia in alloxan-induced diabetic rats.

## 1. Introduction

Hyperglycaemia in diabetes mellitus (DM) is one of the most important factors responsible for the development of oxidative stress, which underlies the major complications in DM patients [1,2]. Oxidative stress is an imbalance between oxidants and antioxidants in favour of the former, potentially leading to cell damage and destruction [3]. The increased generation of reactive oxygen species (ROS) causes damage to cells, tissues and biomolecules, thus contributing to diabetic complications [4]. Dyslipidaemia is common in both insulin deficiency and insulin resistance, which affects enzymes and pathways of lipid metabolism. It is a major risk factor for cardiovascular diseases which is currently a leading cause of morbidity and mortality worldwide [5]. Major metabolic derangements which result from insulin deficiency in type 1 diabetes mellitus (T1DM) are impaired glucose, lipid and protein metabolism [6,7]. Alloxan administration initiates the production of ROS, including superoxide radical (O_2_**^·^**), hydroxyl radical (OH**^·^**) and hydrogen peroxide (H_2_O_2_), which damage, and later destroy, the cells [8]. Although insulin has remained an important component in treatment plans for DM management in patients with type 1 diabetes mellitus (T1DM), it fails to prevent the long-term complications [9]. Therefore, there is urgent need for testing of the potency of probiotics and vitamin C (Vit-C) as effective antioxidants in ameliorating the hyperglycaemia, oxidative stress and dyslipidaemia which accelerate the development of diabetic complications. Probiotics are live microorganisms which, when administered in adequate amounts, confer a health benefit to the host [10]. Several studies have shown that some strains of lactic acid bacteria possess antioxidant properties [11,12]. The antioxidative mechanisms of probiotics may be attributed to ROS scavenging, metal ion chelation, enzyme inhibition, and mitigation of ascorbate autoxidation [11]. Their use as a form of treatment regimen for diseases such as T1DM is currently an area of limited investigation [13]. The vitamin has been shown to play vital roles in DNA repair, reducing the extent of DNA damage [14] and scavenging ROS, caused by oxidative stress [15]. Thus, we hypothesized that Vit-C and probiotic may ameliorate the hyperglycaemia, oxidative stress and dyslipidaemia in DM.

The aim of the present study was to investigate the ameliorative effect of probiotic and Vit-C on hyperglycaemia, oxidative stress and dyslipidaemia of alloxan-induced diabetic rats.

## 2. Materials and Methods

### 2.1. Chemicals

Alloxan was purchased from Sigma Chemical Co. (St. Louis, MO, USA); with Pcode: 101164079. The probiotic, *Saccharomyces cerevisiae* was obtained from Montajat Pharmaceuticals, Biosciences Division, Dammam, Saudi Arabia. Vitamin C 500 mg was obtained from Hubei Tianyao Pharmaceutical Ltd., Xiangyang Hubei, China; while insulin was obtained from Novo Nordisk, A/S, Denmark.

### 2.2. Animal Treatments

Forty-eight (48) male Wistar rats (100–180 g body weight) were obtained from the Vector and Parasitology Unit of the Nigeria Institute for Trypanosomiasis and Onchocerciasis Research (NITOR). They were allowed to acclimatize for two weeks in the Physiology research laboratory of the Department of Veterinary Physiology, Ahmadu Bello University, Zaria, Nigeria, where the experiment was conducted. The rats were housed under standard hygienic conditions in plastic cages with wood shavings as bedding, which was changed every week. Rats were also kept under natural thermal environmental conditions with ambient temperature of 24 °C–26 °C and relative humidity of 70%–80%, and approximately alternating 12 h light/dark cycles. They were given access to a standard pelletized rat chow and water *ad libitum*. The study was approved by the Ahmadu Bello University Committee on Animal Welfare and Use. Also, the animal experiments adhered to the Guide for the Care and Use of Laboratory Animals [16]. Animals were weighed and randomly assigned to six groups and treated as follows:

- *Group 1*, Control group: (normal saline only);
- *Group 2*, Untreated diabetic, UD: (alloxan 150 mg/kg, i.p.);
- *Group 3*, Diabetic + Treated with insulin, DTI: (alloxan 150 mg/kg, i.p. + s.c. injection of insulin 4 U/kg per day);
- *Group 4*, Diabetic + Treated with probiotic, DTP: (alloxan 150 mg/kg, i.p. + *Saccharomyces cerevisiae*, 4.125 × 10^6^ CFU/100 mL, *per os*);
- *Group 5*, Diabetic + Treated with Vitamin C, DTVit-C: (alloxan 150 mg/kg, i.p. + vit-C 100 mg/kg, i.m.);
- *Group 6*, Diabetic + Treated with probiotic and Vit- C, DTPVit-C: (alloxan 150 mg/kg, i.p. + S. *cerevisiae*, 4.125 × 10^6^ CFU/100 mL, *per os*. + Vit-C 100 mg/kg, i.m.).

Experimental animals were treated with insulin, probiotic and Vit-C, after 72 h of verification of diabetes, once weekly for four weeks.

### 2.3. Induction of Diabetes

Diabetes mellitus (DM) was induced in overnight-fasted rats by a single i.p. injection of freshly-prepared alloxan monohydrate, dissolved in a cold physiological saline (0.9% NaCl) solution at the dose rate of 150 mg/kg body weight [17]. The animals were given free access to 5% glucose solution in order to overcome the alloxan-induced hypoglycaemia for the first one hour post-treatment with alloxan. Blood glucose concentration of the rats was estimated 72 h after alloxan administration, and DM was confirmed by analysis of blood samples, collected from the vein at the tip of the tail, using a portable blood glucometer and glucose test strips (On Call^®^Plus, Hannover, Germany). Animals with blood glucose concentration equal or more than 14 mmol/L were considered diabetic and used in the entire experimental group [18].

### 2.4. Measurement of Blood Glucose

Experimental animals were rearranged according to the blood glucose concentration, except the control group before commencement of treatment. Blood glucose concentration in all experimental groups were recorded following 12-h fasting each day, at 8:00 a.m. before feeding the rats, using a portable glucometer (On Call^®^Plus, Hannover, Germany) and glucose test strips.

### 2.5. Measurements of Body Weight and Feed Intake

Rats were weighed individually at weekly intervals using a Mettler Toledo^®^ digital precision weighing balance with a sensitivity of 0.01 g (Model MT-500D), and the body weights were recorded to calculate weekly body weight gains. Feed intake was calculated as the difference between the quantity of feed given and the feed residue left after 24 h of feeding. This was first measured using the 0.01 g Mettler Toledo^®^ digital weighing balance before the morning feeding of the rats.

### 2.6. Collection of Blood

At the end of the experiment, the rats were fasted overnight and killed by jugular venisection after light chloroform anaesthesia. From each rat, 5 mL of blood was collected into dried centrifuge tubes, and allowed to clot at room temperature (24 °C–26 °C). Thereafter, serum was separated from the clot by centrifuging at 2000× *g* for 15 min. The serum was collected in clean bottles and stored at 4 °C until required.

### 2.7. Serum Cholesterol and Triglyceride Assay

Serum was collected by first allowing the blood to clot, followed by centrifugation at 2000× *g* for 15 min. Total cholesterol (TC), high-density lipoprotein cholesterol (HDL-cholesterol) and triglyceride (TG) were determined in serum by colorimetric methods of Allain *et al.* [19] and Burstein *et al.* [20], using enzymatic diagnostic kits (AGAPPE Diagnostic Switzerland GmbH). Low-density lipoprotein cholesterol (LDL-cholesterol) was calculated according to the formula of Friedewald *et al.* [21]: (LDL-chol) = (Total chol) − (HDL-chol) − Triglyceride/5.

### 2.8. Assessment of Lipid Peroxidation of Organs

All organ samples were kept on ice and processed as rapidly as possible. Approximately 10% (*w*/*v*) tissue homogenates were prepared in 10% phosphate-buffered saline (pH 6.4), using a micro-homogenizer (Physcotron, Niti-on Inc., Chiba, Japan). The supernatant was obtained from the homogenate after centrifugation at 5000× *g* for 10 min, and was used in the determination of malondialdehyde (MDA) concentration and enzyme activities. The concentration of lipid peroxidation was estimated in the tissue (brain, kidney, heart and liver) homogenates (10% phosphate-buffered saline). The MDA concentration was measured using the standard method of Northwest Life Science Specialties (NWLSS™) MDA assay kits NWK-MDA01. The method was based on the reaction of MDA with thiobarbituric acid (TBA), forming an MDA-TBA_2_ adduct that absorbed strongly at the wave length of 532 nm [22].

### 2.9. Assay of Antioxidant Enzymes

The activities of superoxide dismutase (SOD), catalase (CAT), and glutathione peroxidase (GPx) were determined using the serum and tissue homogenates of the experimental rats. The activity of SOD (EC.1.15.1.1) was assessed using the NWLSS^TM^ SOD activity assay, which provided a simple, rate method for the determination. The method was based on monitoring the auto-oxidation rate of haematoxylin as described by [23] with modifications to increase robustness and reliability. Briefly, in the presence of SOD enzyme at specific assay pH (8.0), the rate of auto-oxidation was inhibited and the percentage of inhibition was linearly proportional to the amount of SOD present within a specific range. Sample SOD activity was determined by measuring the ratios of auto-oxidation rates in the presence and absence of the sample, and expressed as traditional McCord-Fridovich “cytochrome c” units. The activity of CAT (EC.1.11.1.6) was determined spectrophotometrically according to the method of [24] with modifications to increase robustness and convenience using the NWLSS^TM^ Catalase activity assay kits protocol NWK-CAT01. Activity of glutathione peroxidase (GPx) (EC 1.11.1.9] was measured using the spectrophotometry method [25], based on the Northwest Life Science Specialties (NWLSS^TM^) glutathione peroxidase assay kits protocol NWK-GPX01.

### 2.10. Data Analysis

Values obtained were expressed as mean ± standard error of the mean (Mean ± SEM). Data were analyzed using repeated-measures analysis of variance (ANOVA). Tukey’s post-hoc test was used to compare all treatment groups with the control and between treated groups. GraphPad Windows 4.03 (GraphPad Software, San Diego, CA, USA) was used for the analyses. Values of *p* < 0.05 were considered significant.

## 3. Results

### 3.1. Effects of Treatment with Insulin, Probiotic and Vitamin C on Blood Glucose Concentrations

The blood glucose concentrations obtained are shown in Table 1. In the diabetic treatment groups, blood glucose concentrations decreased significantly (*p* < 0.001) with respect to the final concentrations obtained in the control group. Blood glucose concentration significantly (*p* < 0.05) decreased in the diabetic group treated with probiotic + Vit-C, when compared with that of the untreated diabetic rats. A significant (*p* < 0.05) decrease in blood glucose concentration was obtained in the diabetic group, administered with both probiotic and Vit-C with respect to the diabetic group treated with insulin.

### 3.2. Effects of Treatment with Insulin, Probiotic and Vitamin C on Serum Malondialdehyde Concentrations

**Figure 1:**
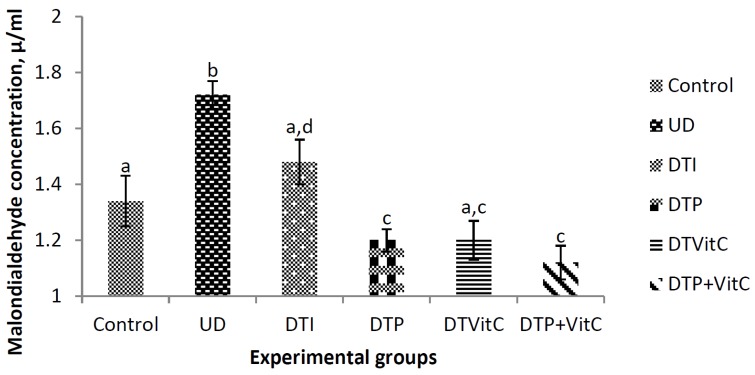
Effects of treatment with insulin, probiotic and vitamin C on serum MDA concentration in alloxan-induced diabetic rats. Abbreviations: UD, untreated diabetic; DTI, diabetic + treated with insulin; DTP, diabetic + treated with probiotic; DTVit-C, diabetic + treated with vitamin C; DTP + Vit-C, diabetic + treated with probiotic and vitamin C. The values are expressed as mean ± S.E. (*n* = 5). Values with superscript letters are significantly (*p* < 0.01) different.

The MDA concentrations were determined in order to evaluate oxidant damage to lipids in all the groups (Figure 1). The MDA concentration in the untreated diabetic group was significantly (*p* < 0.01) higher, when compared with that of the control group. The MDA concentrations in the diabetic groups, treated with a combination of probiotic, Vit-C, and probiotic + Vit-C, were significantly (*p* < 0.001) lower, when compared with the values recorded in the untreated diabetic group or that of the diabetic group treated with insulin.

### 3.3. Effects of Treatment with Insulin, Probiotic, and Vitamin C on the Activities of Serum Antioxidant Enzymes

The difference in the activities of the enzymes between the untreated diabetic group and the controls were insignificant (*p* > 0.05) (Table 2). The activity of SOD in the diabetic group administered with Vit-C was higher (*p* < 0.05), compared with that of the diabetic group treated with insulin. In the diabetic group treated with insulin or probiotic, CAT activity decreased significantly (*p* < 0.05), when compared with that of the control group. In the rats treated with Vit-C or probiotic + Vit-C CAT activity increased significantly (*p* < 0.05), when compared respectively with the groups treated with insulin, and probiotic. The activity of GPx decreased significantly (*p* < 0.01) in the diabetic group treated with probiotic, when compared with the controls. The activity of GPx was higher (*p* < 0.01) in the diabetic group, treated with probiotic and Vit-C combination, when compared with the diabetic group treated with probiotic only.

### 3.4. Effects of Treatment with Insulin, Probiotic and Vitamin C on Brain Malondialdehyde Concentration

**Figure 2:**
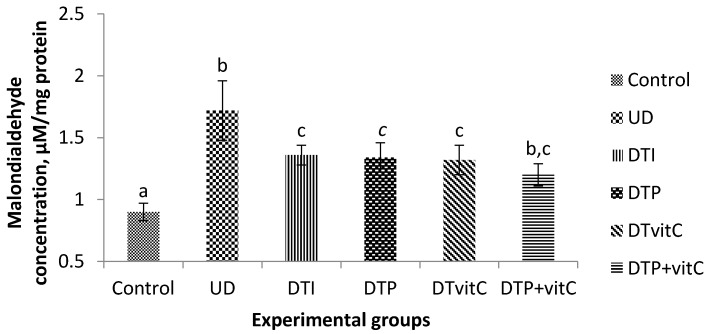
Effects of treatment with insulin, probiotic and vitamin C on brain, malondialdehyde concentrations of alloxan-induced diabetic rats. Abbreviations: UD, untreated diabetic; DTI, diabetic + treated with insulin; DTP, diabetic + treated with probiotic; DTVit-C, diabetic + treated with vitamin C; DTP + Vit-C, diabetic + treated with probiotic and vitamin C. The values are expressed as mean ± S.E. (*n* = 5). Values with different superscript letters are significantly (*p* < 0.01) different.

The concentration of MDA was significantly (*p* < 0.01) higher in the untreated diabetic group, when compared with that of the control group. The differences in MDA concentrations obtained in the diabetic groups, treated with insulin, probiotic, Vit-C and probiotic + Vit-C, when compared with the control group were insignificant (Figure 2).

### 3.5. Effects of Treatment with Insulin, Probiotic and Vitamin C on the Activities of Brain Antioxidant Enzymes

There was a significant (*p* < 0.01) decrease in SOD activity in the untreated diabetic group, when compared with that of the control group (Table 3). Similarly, SOD activity was lower (*p* < 0.01) in the diabetic groups treated with insulin, and probiotic + Vit-C, respectively than that of the control group. A highly significant (*p* < 0.001) increase was recorded in SOD activity in probiotic + Vit-C group, when compared with that of the diabetic group, administered with Vit-C only. The activity of CAT in the untreated diabetic group decreased significantly (*p* < 0.05), when compared with the controls. There was a significant (*p* < 0.001) decrease in CAT activity in the groups treated with Vit-C, and probiotic + Vit-C with respect to the control group. The activity of GPx in the group treated with probiotic only was significantly (*p* < 0.05) higher than that of the controls. The GPx activity was higher (*p* < 0.001) in the diabetic groups treated with Vit-C, and probiotic + Vit-C with respect to the control group.

### 3.6. Effects of Treatment with Insulin, Probiotic and Vitamin C on Kidney Malondialdehyde Concentration

**Figure 3:**
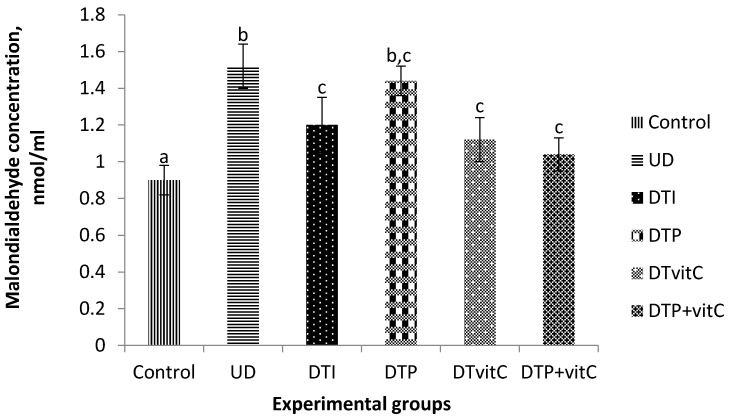
Effects of treatment with insulin, probiotic and vitamin C on kidney malondialdehyde concentrations of alloxan-induced diabetic rats. Abbreviations: UD, untreated diabetic; DTI, diabetic + treated with insulin; DTP, diabetic + treated with probiotic; DTVit-C, diabetic + treated with vitamin C; DTP + Vit-C, diabetic + treated with probiotic and vitamin C. The values are expressed as mean ± S.E. (*n* = 5). Values with different superscript letters are significantly (*p* < 0.05) different.

The effect of treatment with insulin, probiotic and Vit-C on kidney MDA concentration is presented in Figure 3. The concentration of MDA in the untreated diabetic group was significantly (*p* < 0.05) higher than in the controls. The concentration in the diabetic group treated with probiotic was also significantly (*p* < 0.05) higher, when compared with that of the control group.

### 3.7. Effects of Treatment with Insulin, Probiotic and Vitamin C on the Lipid Profile of Alloxan-Induced Diabetic Rats

The results on the lipid profile of alloxan-induced diabetic rats are shown in Table 4. A highly significant (*p* < 0.001) increase in total cholesterol concentration was recorded in the untreated diabetic group, when compared with that of the control.

Conversely, a highly significant (*p* < 0.001) decrease in total cholesterol concentration was observed in the treated groups, when compared with the untreated diabetic group. There was a significant (*p* < 0.05) increase in the triglycerides (TG) concentration of the untreated diabetic group, when compared with that of the control. However, a more significant (*p* < 0.01) decrease in TG concentration was recorded in the diabetic group treated with probiotic + vitamin C, when compared with the untreated diabetic group. Highly significant (*p* < 0.001) reduction in the concentration of high density lipoprotein (HDL) was recorded in the untreated diabetic group, when compared with that of the control. HDL concentration was significantly (*p* < 0.001) elevated in all the diabetic-treated groups, when compared with the untreated diabetic group. There was a highly significant (*p* < 0.001) increase in the concentration of low density lipoprotein (LDL) in the untreated diabetic group, when compared with that of the control. Most importantly, all the diabetic-treated groups recorded a highly significant (*p* < 0.001) reduction in the concentration of LDL, when compared with the untreated diabetic group.

### 3.8. Effects of Treatment with Insulin, Probiotic and Vitamin C on the Body Weights and Feed Intake of Alloxan-Induced Diabetic Rats

The results on body weights and feed intake of alloxan-induced diabetic rats treated with insulin, probiotic and vitamin C are shown in Table 5. There was no significant difference (*p* > 0.05) in the body weights of untreated diabetic group and the treated diabetic groups, when compared with the control group. However, a highly significant (*p* < 0.001) decrease in body weights of the untreated diabetic rats, when compared with that of the control and the treated groups, respectively, was observed. The significant reduction in the body weights of rats in the untreated diabetic group was observed from the second week to the 4th week of the experiment. From week 3, the body weights in all treatment groups were significantly (*p* < 0.001) higher, when compared with the untreated diabetic group.

In week 1, feed intake increased (87.29 ± 7.49 g) in the untreated diabetic group, when compared with the treated diabetic groups (63.57 ± 12.44 g, 80.57 ± 10.44 g, 53.43 ± 10.54 g and 74.43 ± 7.23 g), respectively (Table 5). There was a highly significant (*p* < 0.001) reduction in feed intake in the vitamin C treated group, when compared with that of the control group. Similarly, a significant (*p* < 0.05) reduction in feed intake was observed in the vitamin C-treated group, when compared with the untreated diabetic group. In the second week, feed intake increases in the untreated diabetic group, except for the probiotic-treated group which recorded greater intake. There was a highly significant (*p* < 0.001) decrease in feed intake in the groups treated with vitamin C and probiotic + vitamin C, respectively. Also, in weeks 3 and 4, a highly significant (*p* < 0.001) decrease in feed intake was recorded in the group treated with vitamin C, when compared with that of the control. A more significant (*p* < 0.01) difference in feed intake was observed between vitamin C and the untreated diabetic group.

## 4. Discussion

The results of the present study showed that alloxan at a dose of 150 mg/kg body weight, apparently, caused considerable damage to pancreatic beta-cells to the extent that the secreted insulin was unable to regulate blood glucose, resulting in a significant (*p* < 0.05) increase in blood glucose concentrations [26]. It has been shown that the toxic effect of alloxan in the pancreas is followed by its rapid uptake by the beta-cells and ROS generation [27,28]. In the presence of hydrogen peroxide and Fe^2+^, highly reactive hydroxyl radicals (OH**^·^**) are formed [17]. The results of the present study showed that the hyperglycaemia produced by alloxan apparently induced the over-production of ROS, inactivation of the antioxidant enzymes by the non-enzymatic glycation of proteins and compromised the function of pancreatic beta-cells [29,30]. ROS generation usually leads to cellular damage through several mechanisms (oxidation, interference with nitric oxide (NO) and modulation of detrimental intracellular signalling pathways).

As a form of treatment in order to ameliorate the negative effect of oxidative stress, antioxidant therapy is a very promising intervention in minimizing the complications associated with oxidative stress in diabetes mellitus [31,32]. Thus, the present study showed that Vit-C decreased the blood glucose concentration in diabetic rats, and a more significant lowering-effect was recorded when probiotic and Vit-C were co-administered (Table 1). The blood glucose-lowering effect of Vit-C may be attributed to its inhibition of oxidative stress; that is, Vit-C scavenging of ROS within the aqueous system of the body, protecting protein and DNA from oxidative damage [14]. Hence, Vit-C reduced blood glucose toxicity and prevented damage to beta-cell mass and insulin content. The results of this study are in agreement with previous findings that Vit-C supplementation enhances glycaemic control [33]. Indeed, studies have shown evidence that diabetics have depleted Vit-C and, therefore, require intake levels that are slightly higher than those recommended for non-diabetic individuals [34]. Vit-C is an enzymatic co-factor and antioxidant that is capable of shifting between its oxidized and reduced forms by electron donation. By this mechanistic action, it protects the intracellular membrane, DNA, proteins and lipids against oxidative stress. Advanced glycation end-products (AGEs) are formed by non-enzymatic reaction between glucose and basic amino acids, and AGE levels are directly correlated with serum glucose levels. The mitigation of AGE production by Vit-C may lead to drastic reduction of blood glucose level, thereby reducing hyperglycaemia-induced cell cytotoxicity [34]. Ganesh *et al.* [35] reported that supplementation of vit-C reduces blood glucose level and improves glycosylated haemoglobin in type 2 diabetes. It has also been reported that type 2 diabetic individuals presented reduced fasting glucose and HbA1c levels followed by higher antioxidant activities after consuming yogurt, containing *L. acidophilus* La5 and *B. lactis* Bb12 for six weeks [36].

The results of the present study showed that hyperglycaemia was accompanied with increased lipid peroxidation, as evidenced by the significant increase in MDA concentration in the serum, brain, and kidneys of untreated diabetic rats. The increase in serum MDA concentration in the untreated diabetic rats may be associated with the destruction of erythrocytic membranes and tissues, caused by the oxidative stress (Figure 1). There was apparently greater lipid peroxidation in the brain, as evidenced by the higher MDA level recorded in the untreated diabetic rats (Figure 2). This result is in agreement with the finding that diabetes induces a significant increase in lipid peroxidation of the brain cortex [37]. This finding may be because the brain constitutes one of the most sensitive organs to ROS-mediated damage due to its high oxygen consumption rate, high content of polyunsaturated fatty acids and presence of redox-active metals [38]. The MDA concentration indicates the extent of lipid peroxidation and is used as a biomarker of oxidative stress in cells and tissues [39,40,41,42]. The results of the present study suggest that lipid peroxidation was greater in the untreated diabetic rats, and, for the first time, showed that probiotic and Vit-C administration, especially their combination, to diabetic rats decreased the levels of serum and tissue lipid peroxidation considerably, subsequently restoring the activities of antioxidant enzymes.

Essentially, the results of previous investigators have shown conflicting reports on antioxidant status in T1DM, with reports indicating increased concentrations [43,44,45,46], decreased concentrations [43,46,47], or no change at all [46], depending on the antioxidant investigated. In the present study, low activities of antioxidant enzymes in the untreated diabetic rats were recorded. This finding further supports and thus confirms the previous result that diabetes is associated with impaired antioxidant defences [2]. The depression of the activities of antioxidant enzymes obtained in the present study suggests increased ROS generation in the untreated diabetic rats; and, therefore, increased oxidative stress [48]. The results of the present study corroborate the finding that shows a decline in the activities of antioxidant enzymes and increased MDA concentration in diabetic rats [49]. SOD dismutases superoxide to H_2_O_2_, whereas CAT detoxifies H_2_O_2_ to H_2_O, thereby protecting the tissues from highly reactive OH^·^ [15]. The increased activities of serum SOD and CAT due to Vit-C treatment, apparently, resulted in the amelioration of the toxic effects of the ROS, generated in excess during diabetes. Insulin treatment decreased the blood glucose levels to some extent; but failed to ameliorate the oxidative stress, which is a “hallmark” of hyperglycaemia [9]. Although it has been established that insulin reduces blood glucose concentrations, it does not reverse all the deleterious effects of lipid peroxidation [50]. Maladies of diabetes cannot be totally corrected by insulin treatment; previous work suggested that antioxidant therapy may be an important adjunct treatment regimen for DM [51]. The finding of the present study experimentally demonstrated that the antioxidants, vitamin C and probiotic are beneficial in mitigating the oxidative stress-induced adverse effects, associated with DM. Harisa *et al.* [52] showed a reduction of fasting blood glucose, HbA1c, and MDA concentration in diabetic rats treated with the probiotic *L. acidophilus* alone or in combination with acarbose.

The result of the present study agreed with the finding that in the brain of diabetic rats, decreased inflammatory damage and increased activities of antioxidant enzymes such as SOD occur, and that probiotic and Vit-C treatment suppressed the progression of the oxidative stress [53,54]. Although diabetes considerably decreased the activity of serum SOD as recorded in the present study, which agreed with the work on food regimens supplemented with prebiotics, probiotics and synbiotics that up-regulate SOD-1 [55].

Regarding the lipid profile, it was observed that at the end of the experiment, total cholesterol and triglyceride levels were higher in the untreated diabetic group, and lower in all the diabetic-treated groups. The elevation in serum cholesterol and triglyceride levels induced by alloxan administered at 150 mg/kg body weight may be a result of reduced lipoprotein lipase activity secondary to reduced serum insulin levels. The increase in serum triglycerides corroborates the findings of Elahi-Moghaddam *et al.* [56], who reported significantly high serum triglyceride levels in diabetic rats administered with 135 mg/kg body weight of alloxan. This is contrary to the findings of Kishor *et al.* [57], who reported that alloxan-induced diabetic rats administered with 100 mg/kg body weight, did not show any significant differences in lipoprotein lipid levels compared with non-diabetic controls. This further confirms that it is not only the onset of diabetes but also its severity that has a direct link with the generation of ROS that affects the lipid and lipoprotein profiles. Alterations in serum cholesterol and triglyceride concentrations following the development of diabetes after alloxan or streptozotocin administration to rabbits and rats have been reported [58]. The reduction in high density lipoprotein (HDL) levels in the untreated diabetic rats was highly significant. This may be attributed to the major protein apoB of low-densiy lipoprotein (LDL) which favour hydrolysis of stored triglycerides in the adipocytes thereby releasing them into the circulation leading to a reduction in serum HDL levels [59]. Low HDL and increased triglycerides levels may contribute to the increased risk of cardiovascular disease [60], which is a sequela of dyslipidaemia.

The antioxidants vitamin C and probiotic were able to decrease the levels of cholesterol, triglycerides and low density lipoprotein in diabetic rats. This is consistent with the findings of Saleh [61], who reported that treatment of diabetic rats with two antioxidants (taurine and/or ginseng) induced a significant reduction in serum cholesterol, triglycerides and low density lipoprotein levels. Several *in vitro* studies and *in vivo* trials have provided evidence to support the roles played by probiotics in lowering serum cholesterol and improving lipid profiles. Mechanisms of cholesterol reduction by probiotic via control of cholesterol metabolism have been proposed. One of such mechanisms is the removal of cholesterol by assimilation. The assimilation of cholesterol by probiotics in the small intestine could decrease serum cholesterol by reducing intestinal absorption of cholesterol [62]. Probiotics must be readily viable and growing in order to be able to remove or assimilate cholesterol [63]. Some researchers have suggested that the incorporation of cholesterol into cell membranes could be another mechanism used to reduce cholesterol in media, or that it involves the ability of certain probiotics to deconjugate bile acids enzymatically [64]. In the present study, vitamin C treatment alone, and its combination with probiotic, reduced the cholesterol and triglyceride levels in diabetic rats. This agrees with the findings of Eriksson and Kohvakka [65], who reported that vitamin C improved fasting blood glucose, cholesterol and triglyceride levels of diabetics. This can be ascribed to the ability of vitamin C to prevent low-density lipoprotein (LDL) from oxidative damage and helps in degradation of cholesterol. Secondly, this vitamin is needed by the enzyme cholesterol 7-α-hydroxylase in the first step of bile acid synthesis; by directing cholesterol towards bile acid synthesis it reduces its level in the serum [66]. The increased low density lipoprotein (LDL) level in the untreated diabetic group may be associated with increased generation of ROS. Also, it may be attributed to a diminished number of peripheral LDL receptors or down-regulation of LDL binding to its receptors. However, this requires further investigation. LDL levels were drastically reduced in the diabetic groups treated with probiotic and vitamin C. This was achieved through scavenging of free radicals, thereby decreasing oxidative damage to oxidized LDL. The decrease in LDL level is an indication of a reduction of the risk of coronary heart diseases, which are very common in diabetic patients [5]. Recent studies demonstrated that probiotic bacteria can improve glycaemia and dyslipidaemia [67,68]. All these lend credit to the fact that, when multiple antioxidants are used in combination, they decrease vulnerability to other agents and synergistically potentiate their antioxidant properties [69].

In the present study, there was significant reduction in the body weight of the untreated diabetic rats administered with 150 mg/kg of alloxan. The weight loss may be due to the catabolic processes involved in diabetes mellitus [70,71]. While in the control group, the animals apparently were in a generally good condition, with normal appetite, and progressive weight gain. From the third week of the experiment, body weight in the diabetic treated groups was significantly increased; this may be attributed to the mitigation of catabolic processes by vitamin C and the probiotic. The highest feed intake observed in the untreated diabetic group can be attributed to the deficient levels and/or absence of insulin, which causes an impaired in glucose transport leading to energy deficiency in the cells, resulting to increased feeding to compensate for the energy deficiency [71]. This is in agreement with the findings of Roselino *et al.* [70], who reported increase in feed intake in male Wistar rats administered with 50 mg/kg body weight of streptozotocin.

The results of the present study have shown for the first time that treatment of diabetic rats with probiotic and Vit-C significantly ameliorated hyperglycaemia, lipid peroxidation, dyslipidaemia and increased the activities of serum and tissue antioxidant enzymes. Therefore, adjunct treatment of diabetics with Vit-C and/or probiotic may be beneficial. Further studies on effective and potent antioxidant treatment of DM, involving co-administration of insulin with vitamin C and/or probiotic, should be carried out in order to assess the efficacy of their synergistic effects in counteracting the complications of DM.

## 5. Conclusions

In conclusion, probiotic and Vit-C, by enhancing activities of antioxidant enzymes and reducing MDA concentrations in serum and tissues, effectively ameliorated hyperglycaemia, oxidative stress changes and dyslipidaemia in alloxan-induced diabetic rats.

## Figures and Tables

**Table 1:** Blood glucose concentrations of alloxan-induced diabetic rats treated with insulin, probiotic and vitamin C.

Experimental Groups						
Week	Control	UD	DTI	DTP	DTVit-C	DTP + vit-C
1	4.42 ± 0.71	21.74 ± 2.98 ^a^	19.64 ± 4.15 ^a^	18.20 ± 4.15 ^a^	18.16 ± 3.83 ^a^	17.64 ± 4.33 ^a^
2	4.48 ± 0.66	20.92 ± 2.72 ^a^	19.00 ± 3.62 ^a^	17.60 ± 3.50 ^a^	17.74 ± 3.24 ^a^	16.60 ± 3.56 ^a^
3	4.42 ± 0.71	20.30 ± 2.41 ^a^	21.56 ± 2.43 ^a^	17.60 ± 2.45 ^a^	17.30 ± 2.71 ^a^	15.84 ± 3.04 ^b^
4	4.40 ± 0.64	19.60 ± 2.71	18.10 ± 2.95 ^a^	16.70 ± 2.09 ^a^	16.34 ± 2.23 ^a^	14.80 ± 2.67 ^b^

Abbreviations: UD, untreated diabetic; DTI, diabetic + treated with insulin; DTP, diabetic + treated with probiotic; DTVit-C, diabetic + treated with vitamin C; DTP + Vit-C, diabetic + treated with probiotic and vitamin C; The data are presented as the mean ± SEM (*n* = 5 for each group). Values with superscript letters are significantly (*p* < 0.05) different.

**Table 2:** Effects of treatment with insulin, probiotic and vitamin C on activities of serum antioxidant enzymes of alloxan-induced diabetic rats.

Experimental Groups	SOD (U/mL)	CAT (U/mL)	GPx (µU/mL)
Control	2.34 ± 0.12	49.60 ± 0.75 ^a^	45.60 ± 0.87 ^a,b^
UD	2.20 ± 0.06	47.60 ± 0.68 ^a^	43.80 ± 0.58
DTI	2.06 ± 0.07 ^a^	44.20 ± 1.02 ^b^	42.00 ± 0.32 ^b^
DTP	2.30 ± 0.09	44.20 ± 0.80 ^a,b^	39.80 ± 0.86 ^c^
DTVitC	2.44 ± 0.05 ^b^	49.40 ± 0.51 ^a^	44.60 ± 0.51 ^a^
DTP + Vit-C	2.38 ± 0.07	48.60 ± 1.57 ^a^	45.60 ± 1.63 ^b,c^

Abbreviations: UD, untreated diabetic; DTI, diabetic + treated with insulin; DTP, diabetic + treated with probiotic; DTVit-C, diabetic + treated with vitamin C; DTP + Vit-C, diabetic + treated with probiotic and vitamin C; SOD, superoxide dismutase; CAT, catalae; GPx, glutathione peroxidase. The data are presented as the mean ± SEM (*n* = 5 for each group). Values in the same row with different superscript letters are significantly (*p* < 0.05) different.

**Table 3:** Effects of treatment with insulin, probiotic and vitamin C on the activities of brain antioxidant enzymes of alloxan-induced diabetic rats.

Experimental Groups	SOD (U/mg Protein)	CAT (U/mg Protein)	GPx (U/mg Protein)
Control	2.48 ± 0.10 ^a^	53.40 ± 1.21 ^a^	48.20 ± 0.97 ^a^
UD	2.02 ± 0.08 ^b,d^	52.80 ± 1.77 ^a,b^	49.00 ± 1.64 ^a^
DTI	1.98 ± 0.08 ^b,d^	54.20 ± 1.39 ^a^	47.20 ± 1.39 ^a^
DTP	2.00 ± 0.07 ^b,d^	44.40 ± 1.44 ^b,c^	42.60 ± 0.93 ^b,c^
DTVitC	1.46 ± 0.08 ^b,e^	42.40 ± 1.12 ^b^	38.80 ± 0.73 ^b,c,d^
DTP + VitC	2.30 ± 0.11 ^a,c^	43.20 ± 0.73 ^b^	39.80 ± 0.80 ^b^

Abbreviations: UD, untreated diabetic; DTI, diabetic + treated with insulin; DTP, diabetic + treated with probiotic; DTVit-C, diabetic + treated with vitamin C; DTP + Vit-C, diabetic + treated with probiotic and vitamin C; The data are presented as the mean ± SEM (*n* = 5 for each group). Values in the same row with different superscript letters are significantly (*p* < 0.05) different.

**Table 4:** Lipid profile of alloxan-induced diabetic rats treated with insulin, probiotic and vitamin C.

Parameters	Control	UD	DTI	DTP	DTVitC	DTP + VitC
TC, mmol/L	3.68 ± 0.07 ^a^	6.26 ± 0.05 ^a^	3.80 ± 0.33 ^b^	3.74 ± 0.24 ^a^	3.28 ± 0.09 ^a^	3.28 ± 0.12 ^a^
TG, mmol/L	1.58 ± 0.08 ^b^	2.70 ± 0.07 ^b^	1.56 ± 0.19 ^a,b^	1.60 ± 0.07 ^b^	1.80 ± 0.38 ^b,c^	1.54 ± 0.15 ^b^
HDL, mol/L	1.48 ± 0.09 ^b^	0.48 ± 0.04 ^b^	1.60 ± 0.15 ^b^	1.64 ± 0.15 ^b^	1.32 ± 0.10 ^b,d^	1.42 ± 0.05 ^b^
LDL, mmol/L	2.90 ± 0.10 ^a^	5.52 ± 0.08 ^b^	2.06 ± 0.57 ^b^	1.74 ± 0.63 ^b^	1.06 ± 0.12 ^b,d^	1.38 ± 0.29 ^b^

Abbreviations: UD, untreated diabetic; DTI, diabetic + treated with insulin; DTP, diabetic + treated with probiotic; DTVit-C, diabetic + treated with vitamin C; DTP + Vit-C, diabetic + treated with probiotic and vitamin C; The data are presented as the mean ± SEM (*n* = 5 for each group); Values in the same row having different superscript letters are significantly (*p* < 0.05) different.

**Table 5:** Weekly body weights and feed intake of alloxan-induced diabetic rats treated with insulin, probiotic and vitamin C.

Group		Control	UD	DTI	DTP	DTVitC	DTP+vitC
Body weight, g:	Week 1	127.6 ± 2.86	117.4 ± 8.02	125.6 ± 5.52	120.6 ± 4.55	123.2 ± 7.02	123.4 ± 6.31
	Week 2	140.8 ± 4.81 ^a^	106.6 ± 5.11 ^b^	137.8 ± 2.94 ^b^	125.4 ± 4.24 ^b,c^	128.4 ± 7.23 ^a,c^	129.4 ± 5.37 ^a,c^
	Week 3	150.4 ± 4.07 ^a^	105.0 ± 4.89 ^b,c^	134.8 ± 3.37 ^b^	142.8 ± 4.09 ^a,e^	138.4 ± 7.23 ^a,e^	139.6 ± 5.41 ^a,e^
	Week 4	158.8 ± 5.45 ^a^	103.8 ± 4.75 ^b^	147.4 ± 3.57 ^a,b^	139.4 ± 4.51 ^c^	142.2 ± 7.15 ^c^	142.6 ± 5.30 ^c^
Feed intake, g:	Week 1	118.3 ± 9.25 ^a^	87.3 ± 7.49 ^b,c^	63.6 ± 12.44 ^b,d^	80.6 ± 10.44 ^b,c^	53.4 ± 10.54 ^b,c^	74.4 ± 7.23 ^b^
	Week 2	125.4 ± 8.97 ^a^	98.9 ± 8.82 ^b,c^	89.9 ± 6.08 ^b^	105.7 ± 4.59 ^b^	79.7 ± 5.04 ^b,d^	86.9 ± 5.47 ^b^
	Week 3	123.6 ± 5.67 ^a^	96.4 ± 6.09 ^b,c^	86.3 ± 3.79 ^b^	115.0 ± 5.49 ^a,d^	82.6 ± 5.58 ^b^	93.3 ± 4.78 ^b^
	Week 4	110.0 ± 3.51 ^a^	104.3 ± 5.14 ^a^	84.0 ± 6.96 ^b^	112.4 ± 4.21 ^a^	83.7 ± 6.17 ^b^	104.0 ± 3.70 ^a^

Abbreviations: UD, untreated diabetic; DTI, diabetic + treated with insulin; DTP, diabetic + treated with probiotic; DTVit-C, diabetic + treated with vitamin C; DTP + Vit-C, diabetic + treated with probiotic and vitamin C; The data are presented as the mean ± SEM (*n* = 5 for each group). Values in the same row having different superscript letters are significant (*p* < 0.05).
